# Microwave coprocessing of modafinil with Gelucire^®^: Thermal and compression characteristics

**DOI:** 10.5599/admet.2569

**Published:** 2025-01-19

**Authors:** Derar Omari, Assayed Sallam, Iyad Rashid, Shereen M. Assaf, Faisal Al-Akayleh, Khaldoun A. Al-Sou′od

**Affiliations:** 1 Department of Pharmaceutics and Pharmaceutical Technology, Faculty of Pharmacy, Yarmouk University, Irbid, Jordan; 2 TQ Pharma, Amman, Jordan; 3 Department of Pharmaceutical Technology, Faulty of Pharmacy, Jordan University of Science and Technology, P. O. Box 3030, Irbid 22110, Jordan; 4 Faculty of Pharmacy and Medical Sciences, University of Petra, Amman 11196, Jordan; 5 Department of Chemistry, Faculty of Science, Al Al-Bayt University, P. O. Box 130040, Mafraq, 25113, Jordan

**Keywords:** Tablet compressibility, crystallinity, molecular modeling, carbonyl oxygen of modafinil

## Abstract

**Introduction:**

Modafinil, a wakefulness-promoting agent, is primarily used to treat excessive daytime sleepiness associated with narcolepsy and fatigue. As a BCS class II drug, modafinil exhibits low solubility and high permeability, with its crystalline structure significantly impacting dissolution, bioavailability, and compressibility. This study explores the use of microwave energy to alter the crystalline structure of modafinil in the presence of Gelucire^®^ 48/16, aiming to improve its pharmaceutical properties.

**Methods:**

Modafinil was treated with microwave energy to form complexes with Gelucire^®^ 48/16, and the resulting formulations were compared to hot-melt complexes and physical mixtures. The structural and thermal properties of the complexes were characterized using X-ray powder diffraction (XRPD), differential scanning calorimetry (DSC), and Fourier-transform infrared spectroscopy. Compressibility and compactibility were evaluated through Kawakita model analysis and response surface methodology). The effect of microwaves on molecular interactions was further investigated using molecular modeling.

**Results:**

XRPD analysis revealed distinct crystalline patterns for microwave and hot-melt complexes compared to physical mixtures, with increased amorphousness observed through crystallinity, relative crystallinity, and relative intensity parameters. DSC thermograms indicated a reduction in melting endotherms and heat flow, suggesting structural changes due to complex formation. Compressibility and compactibility studies demonstrated optimal performance at low Gelucire^®^ content, with microwave-treated complexes exhibiting superior properties to untreated mixtures. Molecular modeling confirmed dipole-dipole interactions between modafinil and the hydrophilic portion of Gelucire^®^.

**Conclusions:**

The study demonstrates that microwave energy effectively alters the crystalline structure of modafinil in the presence of Gelucire^®^ 48/16, enhancing its amorphousness, compressibility, and compatibility. These findings highlight the potential of microwave-assisted complexation as a novel approach to improve the pharmaceutical performance of BCS Class II drugs like modafinil.

## Introduction

**Figure 1.**
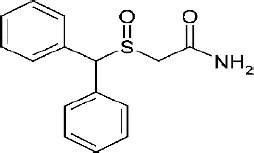
Modafinil chemical structure

Modafinil is a non-amphetamine central nervous system (CNS) stimulant [1] with wakefulness-promoting properties and approved for the treatment of narcolepsy [2], sleep work shift disorder[3], obstructive sleep apnea [4] and a first-line treatment of idiopathic hypersomnia [5,6]. It is a white to off-white crystalline solid practically insoluble in water and cyclohexane and slightly soluble in methanol and acetone. The molecular formula is C15H15NO2S and the molecular weight is 273.4 Da [7]. The chemical structure (Figure 1) possesses a chiral sulfoxide group, making the molecule either the racemic or the R-enantiomer (Armodafinil), which has a longer half-life [8]. The racemic structure has seven different polymorphic forms, of which Forms I, III and IV are more probably investigated. New crystalline forms II-VI of modafinil and processes for preparing them were fully described [9]. Single crystals of (±)-modafinil were grown in a gel medium obtained from the hydrolysis and condensation of tetramethoxysilane (TMOS) [10]. Moreover, the identification and structural characterization of a new metastable polymorphic form, (±)-form IV, of modafinil was obtained through a specific crystallization procedure in TMOS-gel [11]. Modafinil was the subject of many formulation articles in the literature, including oraldispersable tablets [12,13], nanobeads, nanofibers [14], and cocrystals [15].

Study of the crystalline states of drugs, such as polymorphs, solvates, cocrystals, etc., is required during all its development stages to balance physiochemical properties like solubility, stability, dissolution, compressibility and bioavailability [16]. These studies are also crucial since they can prevent the appearance of unexpected solid forms during the industrial processes and after the approval of a drug [17]. Compressibility of drugs [18], polymers [19] or pharmaceuticals in general [20] is one of such properties; different forms of the same crystal show different behavior during compression and so the right crystal form should be selected. It has been reported that the poor compressibility of drug crystals can be attributed to the presence of crystal faces that give poor adhesion to other crystals and the absence of the faces required for optimal adhesion [21,22].

Cocrystals are crystalline materials comprising a drug and a coformer in a single crystal lattice [23]. They can be from molecules of any type possessing bonding functional groups, held together by non-covalent interactions like hydrogen bonding, van der Waals forces and π-π interactions. Cocrystal formation is considered an effective method to improve the aqueous solubility of poorly soluble drugs [24]. Cocrystals can be prepared using solvent- and solid-based methods. The solvent-based methods involve slurry conversion solvent evaporation, cooling crystallization and precipitation. The solid-based methods involve net grinding, solvent-assisted grinding and sonication [25]. Therefore, the crystallization method determines the product properties, which can be modified by selecting proper crystallization conditions. In this regard, microwave irradiation was investigated to achieve such a purpose [16,26].

Microwaves (MWs) are electromagnetic waves with a wavelength in the range of 0.001 to 0.3 m, shorter than that of a normal radio wave but longer than those of infrared radiation [27]. MWs can affect matter in two different ways: first, by thermal effect due to dipolar polarization and ionic conduction [28] and second, by electrostatic polar effects which lead to dipole-dipole type interactions between the dipolar molecules and the charges in electric field [29,30]. MW heating possesses many advantages over conventional heating, including rapid heating and cooling, reduced temperature gradients across the sample, lower energy practice and enhanced reaction rates [31]. In the MW technique, the heat is generated inside the material and then passes to the entire volume with a constant heating rate. MWs have the ability to penetrate any material, leading to heat production everywhere in the material at the same time [32]. Amorphization via the formation of amorphous solid dispersion (ASD) and co-crystallization are two approaches utilizing MWs to modify drug properties such as solubility, dissolution rate, compressibility, etc. In this context, Pagire *et al.* [33] explored the microwave-assisted synthesis of caffeine/maleic acid cocrystals, emphasizing the role of the dielectric and physicochemical properties of the solvent. Similarly, Hempela *et al*. [34] investigated microwave-induced in situ drug amorphization using a mixture of polyethylene glycol and polyvinylpyrrolidone. Furthermore, Holm *et al*. [35] developed a multiparticulate drug delivery system for *in situ* amorphization. In a related study, Madan *et al.* [36] prepared, characterized, and evaluated tablets containing microwave-assisted solid dispersions of apremilast in vitro. Additionally, Holm *et al*. [37] studied microwave-induced in situ amorphization facilitated by crystalline hydrates

Microwave induction on modafinil as an active pharmaceutical ingredient has been previously carried out to enhance the drug’s solubility and bioavailability [38]. However, when microwave processing has been thoroughly investigated, particularly in the case of modafinil, it was found that such technique was mostly carried out in the presence of a complexing agent or a carrier forming a solid dispersion system. Trimethyl chitosan and poly(vinylpyrrolidone) [39], and PEG [40] were reported examples of such agents.

In search for an effective carrier that manifests enhanced drug solubility and bioavailability when subjected to microwave processing, Gelucire^®^ and its derivatives were a favorite choice in solid dispersion. Atorvastatin calcium [41], paracetamol [42], mefenamic acid and flufenamic acid [43], and ibuprofen [44] are some examples of APIs involved in microwave-processed formulations that used Gelucire^®^ as a carrier. In fact, as stated by the manufacturer, Gelucire^®^, as a non-ionic surfactant, can be used as a lubricant and a solubility enhancer in pharmaceutical formulation [45].

However, in addition to its solubility shortcoming, it has to be emphasized that modafinil fails to be processed in direct compression tableting. In its powder state, the processing of modafinil into a solid dosage form has been subjected to wet granulation [46], dry granulation [47], and wet mixing [48]. Generally, a powder with poor powder flow, low compressibility and compactibility necessitates wet granulation in many pharmaceutical applications [49]. Nevertheless, there were reported attempts to directly compress modafinil processed through the sublimation technique of different terpenes. However, there was no compression analysis to emphasize the improvement in powder and tablet physical properties [50].

Based on the aforementioned shortcoming, the current research intends to investigate a comprehensive compression analysis of the modafinil/Gelucire^®^ 48/16 system irradiated with microwaves. In parallel, a hot-melt technique involving the same drug-Gelucire^®^ complex will be carried out for comparison purposes. Experimental work will be set through experimental design to identify the optimum Gelucire^®^ concentration that manifests the maximum tablet desired physical properties and maximum compression parameters. The foregoing will be evaluated using the Kawakita model of compression analysis. The modafinil-Gelucire^®^ complex will be characterized using XRPD, FTIR and DSC analysis. Finally, the identity of complex formation will be investigated for any inter-molecular interaction through molecular modeling.

## Materials and methods

### Materials

Modafinil (USP, Batch No 1602003257 by Alembic, polymorph I) and Gelucire^®^ 48/16 are kind gifts from TQ Pharma, Amman, Jordan. Acetone (pharmaceutical grade) was purchased from a local vendor.

### Preparation of microwaved samples

Modafinil-Gelucire^®^ cocrystals were prepared as per the design in Table 1. Modafinil and Gelucire^®^ in the appropriate weight were sieved in a 250-micrometer sieve before dissolving in 500 ml of acetone in a 45 °C water bath. Samples were then dried with a 30 % power Microwave (Domestic Microwave, SHARP Co. China) and placed in a laboratory hood for 30 min. Dry samples were collected and kept in appropriate containers until used.

**Table 1.** Levels of the 13 runs and their corresponding mass contents for the microwaved modafinil-gelucire^®^ samples.

Code	-1	-0.75	-0.67	-0.5	-0.34	-0.25	0	0.25	0.34	0.5	0.67	0.75	1
Content of Gelucire^®^, wt.%	0.0	2.5	3.4	5.0	6.7	7.5	10.0	12.5	13.4	15.0	16.7	17.5	20.0

### Preparation of hot-melt samples

Modafinil and Gelucire^®^ complexes were prepared per the design in Table 2. Modafinil and Gelucire^®^ were sieved by a 250-micrometer sieve. Gelucire^®^ was melted in a 60 °C water bath, mixed very well with modafinil powder, and removed from the water bath until congealed. Samples were collected and kept in appropriate containers until used.

**Table 2.** Levels of the 5 runs and their corresponding mass contents for the hot-melted modafinil-gelucire^®^ and physical mixtures samples.

Code	-1	-0.5	0	0.5	1
Content of gelucire, wt.%	0	5	10	15	20

### Preparation of physical mixtures samples

The specified amount of samples (Table 2) were dissolved in 500 mL acetone and kept in a hood under air flow until complete drying. Powder samples were collected and kept in appropriate containers.

### Response surface methodology analysis

Mixtures handled using the RSM technique were subjected to one-factor design using Design-Expert^®^ software version 11. The analysis used a quartic model based on a fractional factorial design of 13 runs for the microwaved tests and 5 for hot-melted ones. The response surface plots were generated to see how responses (*a*, *P_K_*) vary with Gelucire^®^-excipient-mass content (*a*, *P_K_* represent compression parameter, which will be discussed shortly). Five lack-of-fit points and 5 replicate points will be chosen for optimal Integrated Variance design that provides lower average prediction variance across the whole range of excipient mass contents. Tables 1 and 2 below present the levels and their corresponding mass contents. Two types of samples comprising 13 and 5 runs were tested; these samples were subjected to processing either by microwave (Table 1) or by hot-melt (Table 2) methods, respectively.

### Compression analysis

The compression behavior of the samples was evaluated using the Kawakita model of powder compression. The Kawakita equation (Equation (1)) takes into account the extent of powder volume reduction (*C*) when a force (*P*) is applied to the powder bed inside the die [51,52].



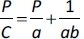

(1)


Two Kawakita parameters were considered from this equation: constant *a* is the minimum porosity of the powder bed before compression, and *P*_K_ (or 1/*b*) is the pressure required to reduce the powder by 50 % of the initial height. The second parameter relates to the strength of the granules and, hence, their ability to resist the applied pressure. It is worth noting that the extent of compressibility can be best interpreted by the first parameter (*a*).

Prior to the compression of modafinil-Gelucire^®^ samples from either of the two aforementioned techniques, powder bulk density was measured. After tablet ejection, tablet thickness was measured using a digital caliber. Compression was carried out using the Gamlen Tablet Press or GTP (Gamlen Tableting Ltd., Biocity Nottingham, UK), whereby 150 mg of the samples were used. Compression forces were set at 100, 200, 300, 400 and 500 kg using a punch size of 6.0 mm diameter at a speed of 60 mm min^-1^.

Volume reduction (*C*) is calculated by Equation (2):



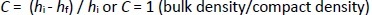

(2)


where *h*_i_ and *h*_f_ represent the initial and final heights, respectively. Compact density is calculated by dividing the tablet mass (150 mg) by the tablet volume. Tablet volume is calculated by multiplying tablet surface area by *h*_f_.

### Fourier transform infrared spectroscopy

FTIR spectra were obtained using an FTIR spectrometer (Bruker, Billerica, MA, USA) with potassium bromide (KBr) pellets. The samples of modafinil, Gelucire^®^ and their complex preparations were scanned from 4000-400 cm^-1^. The resultant spectra were recorded.

### X-ray powder diffraction -crystallinity analysis

X-ray powder diffraction (XRPD) patterns for modafinil pure material and modafinil-Gelucire^®^ complex preparations were obtained using a Philips X-ray powder diffractometer (PW 1729, Netherlands). Samples were scanned over 2*θ* range of 5 to 70°.

Three main parameters associated with X-ray diffraction analysis were given a great deal of interest in this work: crystallinity, relative crystallinity (*RC*), and relative intensity (*RI*).

Initially, crystallinity was calculated by dividing the amount of the crystalline phase (or summation of all peak area) by the total amounts of crystalline and amorphous phases, and then the result was multiplied by the concentration of modafinil inside the complex [53,54]. Practically, it is equal to: [summation of all peak areas + summation of areas for the baseline for the whole 2*θ* range excluding peak areas] x modafinil concentration.

Relative crystallinity (*RC*) for each peak and relative intensity (*RI*) were calculated by dividing the area and intensity of individual diffraction peaks, respectively, by the highest and/or most robust diffraction peak of the raw modafinil sample manifested at a 2*θ* value of 17.87°. Total relative crystallinity (total *RC*) was calculated by summing up *RC* areas for all diffraction peaks, whereas the average relative intensity (average *RI*) was calculated by summing all RIs and then dividing the result by the number of peaks.

### Differential scanning calorimetery

The DSC thermograms of modafinil, its complexes and physical mixtures were recorded in a differential scanning calorimeter (DSC 821; Mettler Toledo AG, Giessen, Germany) in nitrogen (flow rate: 50 mL min^-1^) at a heating rate of 10 °C min^-1^ in the range of 20-220 °C. A 4.0 mg (± 0.1) mass was weighed in a hermetically sealed aluminum crucible. The DSC was calibrated by indium.

### Molecular modeling

In order to elucidate the intermolecular interactions and calculate the interaction energies between modafinil and Gelucire^®^ 48/16, molecular modeling techniques were employed. Molecular modeling (MM) investigations were carried out in water to forecast the generation of modafinil/Gelucire^®^ complexes and to measure their binding affinity employing Hyperchem^®^ (release 8.06). Force field simulation packages used in these computations were Amber94, enhanced MM, BIO+ (CHARMM) and optimized potential for liquid formulation (OPLS) method actualized in Hyperchem^®^ using the atomic charges or bond dipoles options for calculating electrostatic interactions. Bond, angle, torsion, non-bonded, electrostatic and hydrogen-bonded interactions were calculated for all force fields. Partial atomic charges were achieved through AM1 semi-empirical calculations and the amount of charge on each atom (total number of modafinil and Gelucire^®^ atoms were 34 and 80, respectively) was assigned.

**Figure 2.**
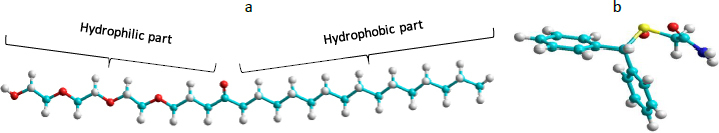
Chemical structure of (a) Gelucire^®^ 48/16 and (b) modafinil

Energy minimizations were achieved employing the Polak-Rebiere algorithm (4.18 kJ mol^-1^ nm^-1^) gradient. The Amber force field was employed to further optimize these geometries by restricting the dihedral angles and the average values. Modafinil was developed from natural bond angles as defined in this software. The structures (Figure 2) were then minimized with the MM, Amber, BIO+ (CHARMM) and OPLS force field, and the resulting structure was further optimized at the HF-ab initio level with the 3-21G basis set (310 basis functions were used with 507 primitive Gaussians).

## Results

### Compression analysis

The straight-line intercepts and slopes of the data generated from Kawakita analysis for *P*/*C* versus *P* are presented in Tables 3 and 4 for the microwave and the hot-melt samples, respectively.

**Table 3.** Kawakita parameters *a* and *P*_*K*_ calculated from the slopes and intercepts of the Kawakita plots for the microwave modafinil-Gelucire^®^ samples. Tablet hardness is also presented.

Code	Slope	Intercept	*a*	*P*_k_	Tablet hardness, N
-1.00	1.1656	10.989	0.857927	9.427763	Fragile
-0.75	1.1345	13.640	0.881446	12.02292	
-0.67	1.1772	21.013	0.849473	17.84998	40-45
-0.50	1.2363	6.3602	0.808865	5.144544	Fragile
-0.34	1.1608	4.725	0.861475	4.070469	
-0.25	1.1661	10.933	0.857559	9.375697	
0.00	1.1788	10.007	0.848320	8.489141	
0.25	1.1922	19.060	0.838785	15.98725	
0.34	1.1812	15.041	0.846597	12.73366	
0.50	1.2420	6.5668	0.805153	5.287279	
0.67	1.1702	11.434	0.854555	9.770979	
0.75	1.1729	10.273	0.852588	8.758632	
1.00	1.1810	15.273	0.84674	12.93226	

**Table 4.** Kawakita parameters *a* and *P*_k_ calculated from the slopes and intercepts of the Kawakita plots for the hot-melt modafinil-Gelucire^®^ samples. Tablet hardness is also presented

Code	Slope	Intercept	*a*	*P_K_*	Tablet hardness, N
-1.00	1.0129	1.8836	0.987264	1.850611	Fragile
-0.50	0.995	9.1184	1.005025	9.164221	30-35
0.00	1.0049	1.314	0.995124	1.307593	Fragile
0.50	1.0143	1.1579	0.985902	1.141575	
1.00	1.0026	1.2717	0.997407	1.268402	

**Figure 3.**
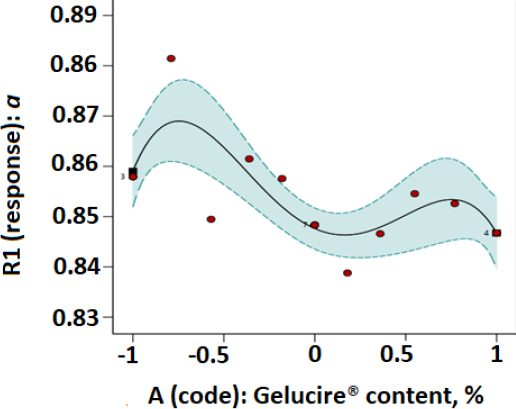
RSM plot of microwaved modafinil-Gelucire^®^ for a one-factor design with *a* as the Kawakita parameter.

**Figure 4.**
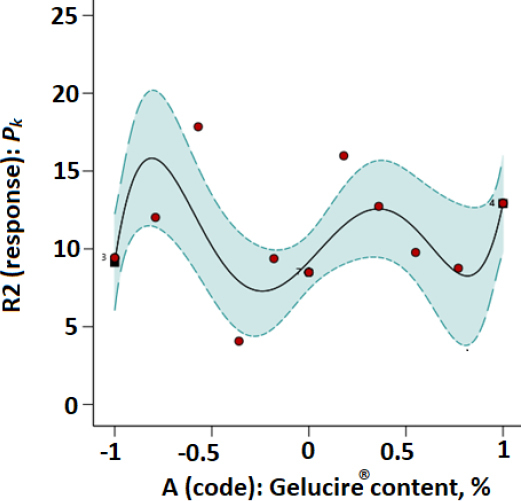
RSM plot of microwaved modafinil-Glucire for a one-factor design with *P*_K_ as the Kawakita parameter.

**Figure 5.**
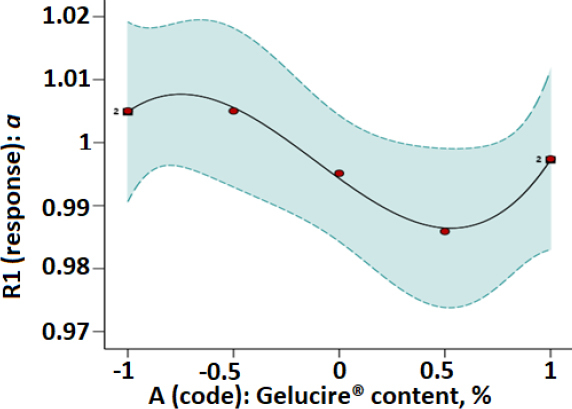
RSM plot of hot-melt modafinil-Gelucire^®^ for a one-factor design with *a* as the Kawakita parameter.

**Figure 6.**
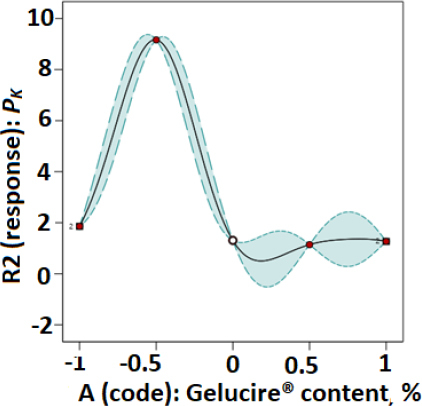
RSM plot of hot-melt modafinil-Gelucire^®^ for a one-factor design with *P*_K_ as the Kawakita parameter.

Plots of parameters *a* and *P_K_* versus modafinil-Gelucire^®^ concentration data designed based on the RSM technique are presented in Figures 3-6 for the microwave (Figures 3 and 4) and the hot-melt (Figures 5 and 6) samples. All plots below were best fitted using a quartic model whereby, statistically, model terms were significant since *p*-values for each plot were <0.05.

Tables 3 and 4 indicate that at low Gelucire^®^ concentrations, Kawakita parameters *a* and *P*_K_ were optimum for the microwave and hot-melt modafinil-Gelucire^®^ samples. This means high powder compressibility and maximum granule strength are attained at such low Gelucire^®^ concentration in the modafinil-Gelucire^®^ matrix. It can be noticed that code -0.67, or a Gelucire^®^ content of 3.4 wt.%, manifested the highest powder compressibility (*a*) and the highest granule strength (*P*_K_) for microwave samples (Table 3). Similarly, at a low Gelucire^®^ content of 5 wt.% or a code of -0.5, compressibility and granule strength were maximized for the hot-melt samples.

**Figure 7.**
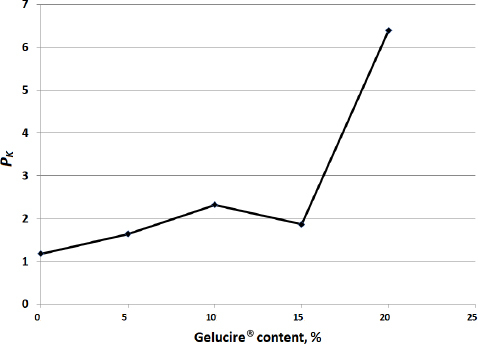
Kawakita *P_K_* values for physical mixtures comprising modafinil and Gelucire^®^ components.

**Figure 8.**
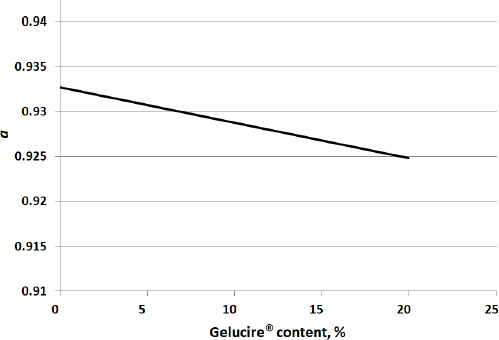
Kawakita *a* values for physical mixtures comprising modafinil and Gelucire^®^ components.

For comparison purposes, physical mixtures comprising modafinil-Gelucire^®^ components were prepared without subjecting the mixtures to microwave and hot-melting processing. Gelucire^®^ was physically mixed with the drug. Kawakita *P*_K_ and *the* results are presented in Figures 7 and 8, respectively. Physically mixed samples showed low *P*_K_ (Figure 7) and *a* (Figure 8) values compared to samples subjected to microwave processing. At only 20 % of Gelucire^®^, the *P*_K_ value was presented the highest, though the foregoing remains much lower than that of microwave samples.

### X-ray powder diffraction analysis

**Figure 9.**
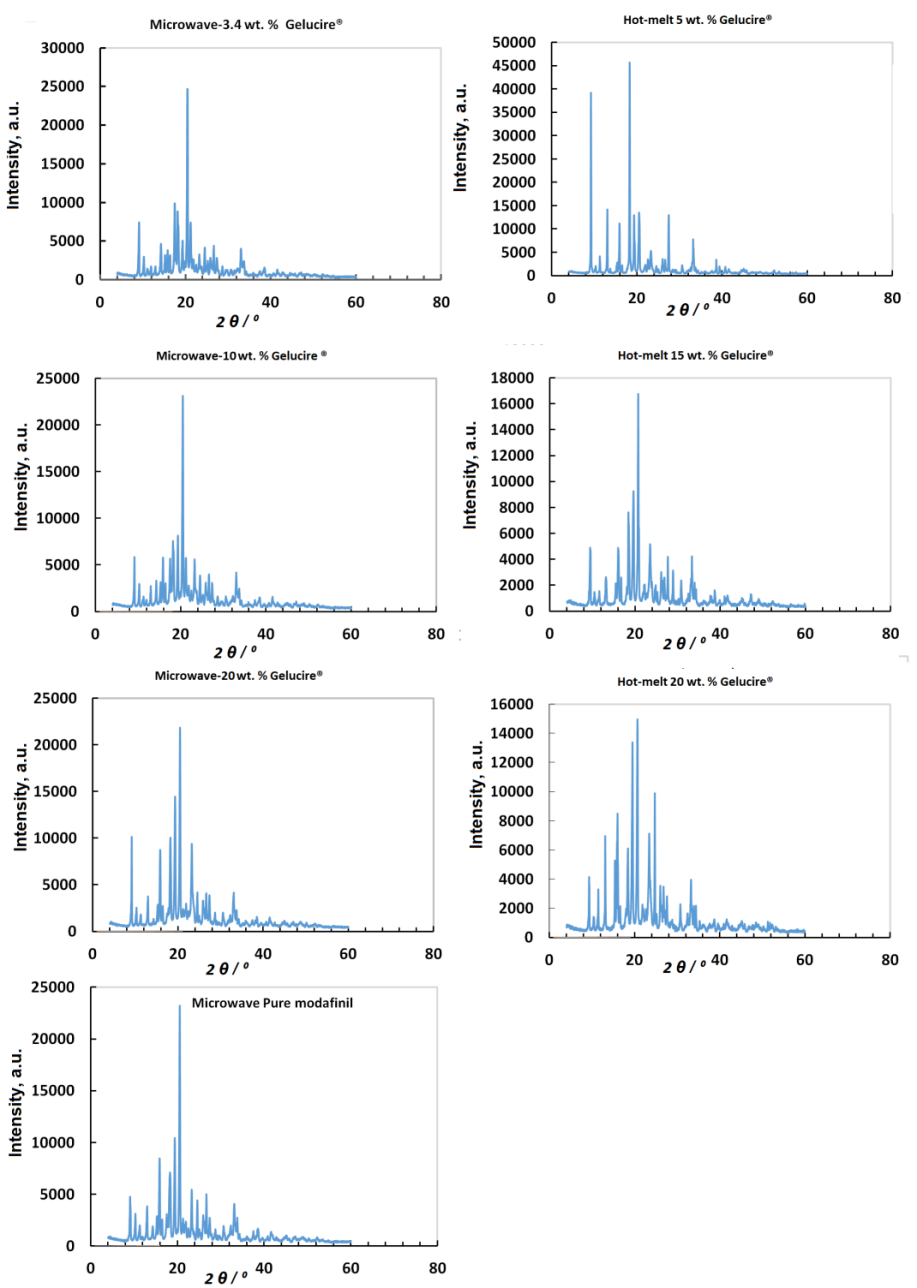
X-ray patterns of modafinil-Gelucire^®^ complexes processed through microwave and hot-melt techniques.

X-ray results for the modafinil-Gelucire^®^ complex subjected to microwave and hot-melt processing in addition to the pure drug are presented in Figure 9. From the Figure, crystallinity values for modafinil/Gelucire^®^ physical mixtures, hot-melt and microwave samples were calculated. Results are presented in the 1^st^ column of Table 5. In parallel, *RC* and *RI* were recorded for each sample and presented in the 2^nd^ and 3^rd^ columns of Table 5, respectively. In this regard, it was desired to investigate changes -if present- in complex crystallinity due to the type of process induced upon complex formation (hot-melting or microwave processing). These changes and variations in Gelucire^®^ content within the modafinil-Gelucire^®^ complex were concurrently considered.

**Table 5.** Crystallinity, relative crystallinity (RC) and relative intensity (RI) values for modafinil-Gelucire^®^ mixtures processsed through hot-melt or microwave techniques. Parameters of physical mixtures were included for comparison purposes.

Sample type	Crystallinity	*RC*	*RI*
Physical mix -0.5 (5 % Gelucire^®^)	71.904	8.161	1.077
Physical mix 1 (20 % Gelucire^®^)	54.852	8.120	0.884
Hotmelt -0.5 (5 % Gelucire^®^)	58.148	9.335	0.727
Hotmelt 0.5 (15% Gelucire^®^)	56.624	7.618	0.509
Hotmelt 1 (20 % Gelucire^®^)	59.116	7.599	0.803
Hotmelt -1 (Pure modafinil)	54.382	9.667	1.913
Microwave -0.67 (3.4 % Gelucire^®^)	42.479	6.210	0.707
Microwave 0 (10 % elucire)	48.166	7.376	0.655
Microwave 1 (20 % Gelucire^®^)	55.005	8.522	0.964
Microwave -1 (Pure modafinil)	39.261	5.318	0.566
Pure modafinil	51.832	7.496	0.860

Crystallinity was calculated based on either the summation of areas/intensities or based on intensities of individual peaks to the most robust diffraction peak of the raw material that did not undergo major changes upon processing. All of the aforementioned parameters were multiplied by the modafinil concentration inside the complex. This algebraic step was crucial since all crystallinity parameters will consider a quantitative contribution of modafinil based upon its content within the complex. Therefore, % crystallinity, *RC* and *RI* parameters will consistently correspond to factors affecting crystallinity as per Gelucire^®^ amount for the modafinil-Gelucire^®^ complex.

### Differential scanning calorimetry analysis

**Figure 10.**
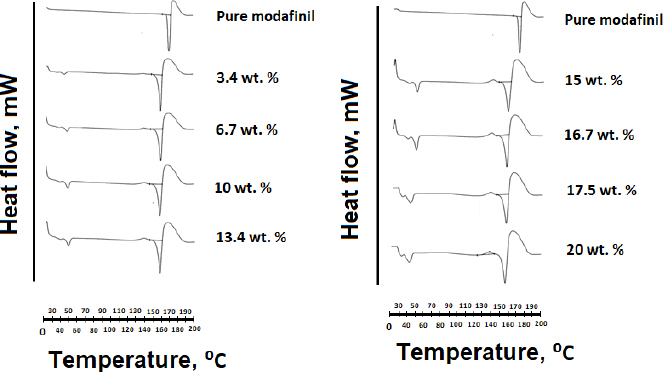
DSC thermograms of modafinil-Gelucire^®^ complex samples subjected to microwave processing at different concentrations of Gelucire^®^ contents.

**Figure 11.**
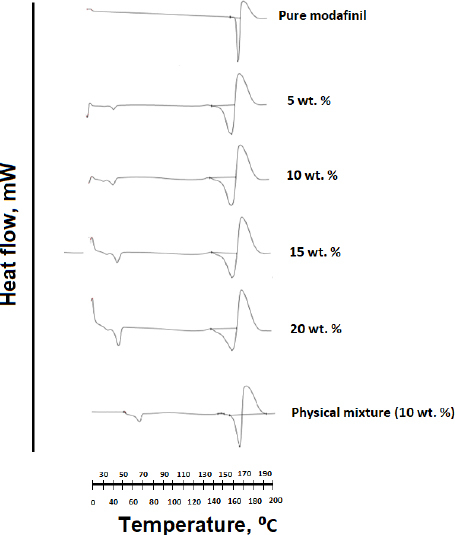
DSC thermograms of modafinil-Gelucire^®^ complex samples subjected to hot-melt processing at different concentrations of Gelucire^®^ contents. A 10 % Gelucire^®^ physical mixture with modafinil was used for comparison.

Differential scanning calorimetry (DSC) thermograms for the pure drug, microwaved, and hot-melt samples are presented in Figures 10 and 11, respectively. For the case of pure modafinil drug, a melting endotherm was detected through a sharp peak at 172 °C. The position of this peak was found to undergo shifting to lower temperatures for both hot-melt and microwave samples except for the physical mixture sample in Figure 11. It is worth mentioning that at each Gelucire^®^ content, a lower melting point for samples subjected to microwave processing was noticed upon comparison with samples subjected to hot-melt processing (Table 6).

**Table 6.** Melting points and melting enthalpy of pure modafinil and modafinil/Gelucire^®^ complexes under microwave and hot-melt processing

Microwave			Hot-melt		
Gelucire^®^ content, wt.%	Melting point, °C	Melting enthalpy, J g^-1^	Gelucire^®^ content, wt.%	Melting point, °C	Melting enthalpy, J g^-1^
0.0	168.9	111.9	0	168.9	111.9
3.4	159.9	63.4	5	164.5	65.0
6.7	159.0	66.7	10	161.9	66.3
10.0	158.7	39.2	15	160.9	51.4
12.5	157.5	42.8	20	160.4	46.9
13.4	157.7	39.4			
15.0	156.8	37.8			
16.7	39.2	156.3			
17.5	156.4	30.9			
20.0	154.9	27.3			

Another important remark associated with DSC thermogram data analysis was the calculated melting enthalpy at every Gelucire^®^ content for hot-melt and microwave samples. Table 6 illustrates the melting enthalpy for both processes based on modafinil melting thermogram areas. It is clear that the enthalpy generated for melting hot-melt samples is higher than that generated for melting microwaved samples.

Based upon the profound changes in x-ray properties of the modafinil-Gelucire^®^ complex compared to the pure drug, it is thought there could be a possible interaction between the two components. This was investigated by testing changes in the FTIR spectra of the complex and by carrying out a molecular modeling (MM) exercise.

### Fourier transform infrared spectroscopy analysis

**Figure 12.**
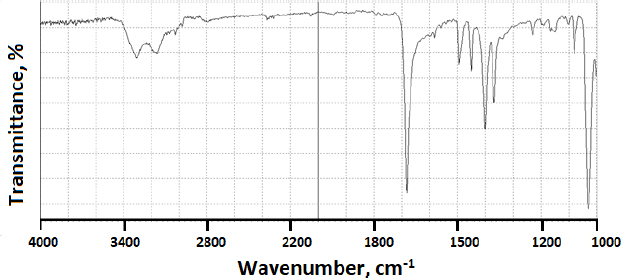
FTIR spectrum of pure modafinil

FTIR was initially tested on modafinil as a pure powder compound. The infrared identity of the compound is presented in Figure 12, whereby Table 7 summarizes C-C, C-H, N-H, C-N, and S=O bond types and their corresponding wavenumbers [55].

**Table 7.** Modafinil bond types and their corresponding wavenumbers.

Wave number, cm^-1^	Bond Type
3550-3330	N-H asymmetric stretching vibration
3450-3250	NH_2_ symmetric stretching vibration
1630-1610, 1090-1060, 700-500 and <500	NH_2_ bending vibrations
1650-1200	C-C stretching vibrations
1382-1266	C-N stretching vibrations
1065-1030	S=O stretching vibrations of sulfoxides

**Figure 13.**
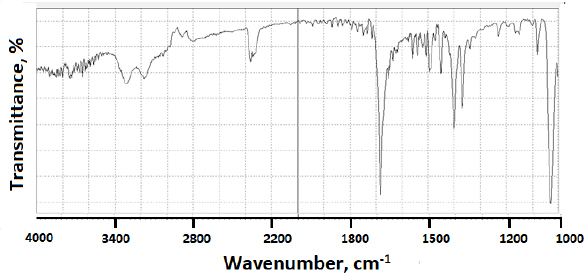
FTIR spectrum of microwaved pure modafinil.

**Figure 14.**
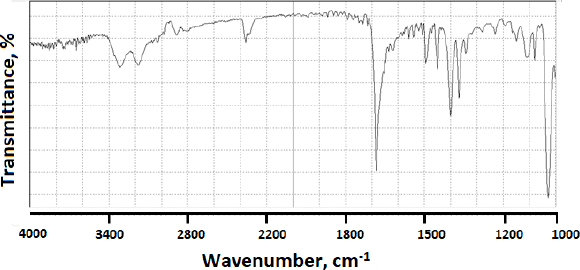
FTIR spectrum of microwaved modafinil with 10 % Gelucire^®^

**Figure 15.**
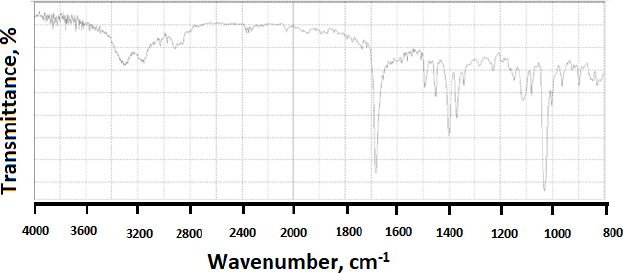
FTIR spectrum of hot-melted modafinil.

**Figure 16.**
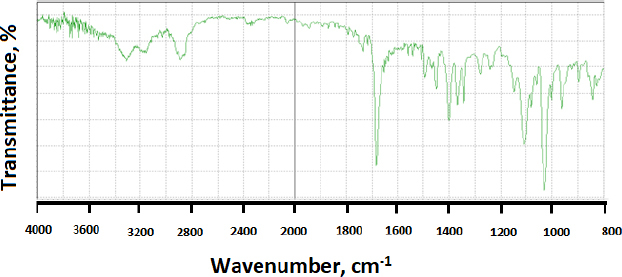
FTIR of hot-melted modafinil with Gelucire^®^ (10 wt.%)

When modafinil was microwaved or hot-melted in the absence or presence of Gelucire^®^ at 10 wt.% content, *i.e.*, in Figures 13 and 14 for microwave and Figures 15 and 16 for hot-melt samples, respectively, the main identity peaks shown in Table 7 remained intact without any displacement after modafinil was being processed. The foregoing is typically valid for both microwave and hot-melt processing techniques. However, Figures 13-14 illustrate the appearance of new low-intensity peaks between 1500-1550 cm^-1^. In addition, Figures 13-14 showed that the baseline of modafinil, when subjected to microwave processing, became highly noisy, unlike the smooth baseline manifested by pure modafinil.

For the hot-melt sample, the appearance of the new peaks between 1500 to 1550 cm^-1^ was less likely than in microwave samples. Similarly, the baseline for hot-melt samples was less noisy compared to that for microwave samples.

**Figure 17.**
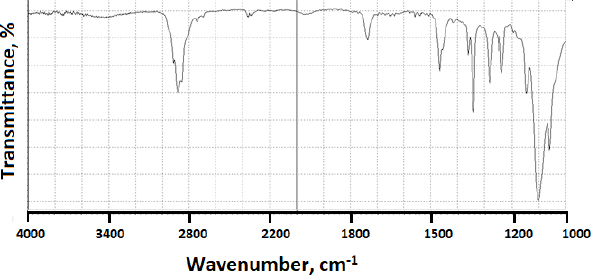
FTIR spectrum of Gelucire^®^.

Lastly, the main identity peak of Gelucire^®^ presented in Figure 17 did not show a remarkable contribution to FTIR data analysis due to the dilution effect upon processing with modafinil.

### Molecular modeling investigation

Two different models were constructed: **Model 1 -** Modafinil molecule was allowed to approach near hydrophobic part in Gelucire^®^ polymer. The complex was optimized using semi-empirical calculations (AM1, 0.1 gradient). Then, the resulting complex was optimized using amber and MM+ (atomic charge and bond dipoles) Amber, BIO+ (CHARMM) and OPLS force fields. **Model 2 -** Modafinil molecule was allowed to approach the near hydrophilic part in Gelucire^®^ polymer, and the resulting complex was optimized as described in Model 1.

The binding energies in both models were estimated and tabulated in Table 8 utilizing Eqation (3):



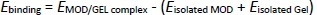

(3)


**Table 8.** Free energy calculations for different models of complexation (Models 1 and 2)

Model	*E*_binding_ / kJ mol^-1^				OPLS
	MM (bond dipole)	MM (atomic charge)	Amber99	BIO+	
1	-33.01	-26.44	-26.32	-25.98	-24.77
2	-71.34	-43.18	-59.20	-44.85	-44.81

Model 2 was found to be more favored than model 1 in all complex cases.

**Figure 18.**
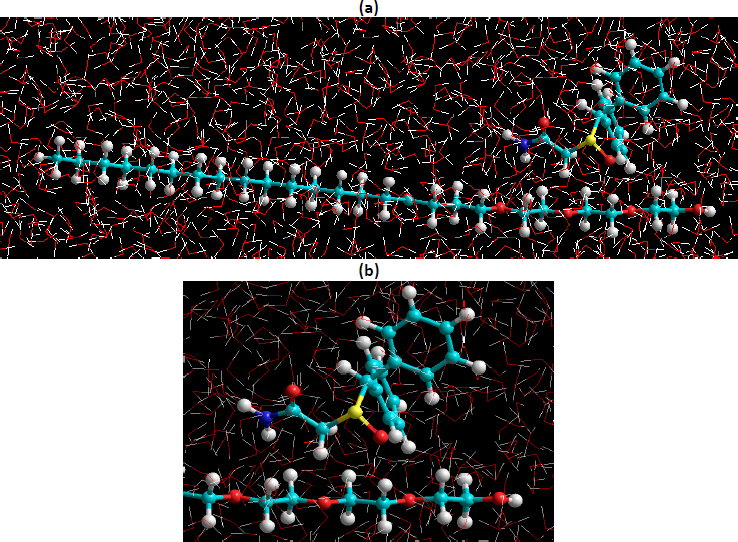
The most favorable 1:1 Modafinil/ Gelucire^®^ complex in water using MM (bond dipole) force field. (a) complete structure of modafinil and Gelucire^®^ (b) magnified image of the part of the structures where interaction is supposed to occur

The binding energy of the most stable complex (Figure 18) was found to be -71.05 kJ mol^-1^ for MM (bond dipole) (Table 8), indicating that complex formation is thermodynamically favored.

**Figure 19.**
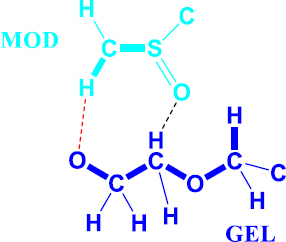
Hydrogen bonding interactions between modafinil/Gelucire^®^. Atoms not connected from both modafinil and Gelucire^®^ are omitted for clarity.

In model 2, both Modafinil/(hydrophilic) Gelucire^®^ interact *via* non-classical hydrogen bonding, namely, (modafinil) C—···O = C (Gelucire^®^) [D···A distance = 0.261 nm and D—···A angle= 41.3°; D = donor and A = acceptor] and (Gelucire^®^) C—···O = S (modafinil) [distance 0.250 nm and angle 31.1°], in a concerted way forming an assembled cyclic motif composed of (modafinil)C—···O—C—C—O—C—···O=S (modafinil) skeleton, Figure 19

The calculated binding energies using all MM force fields suggest that the modafinil/Gelucire^®^ complexes formation is thermodynamically favored (Table 8). Based on these binding energies, it is obvious that the most stable complex was for the model 2 complex, in which the modafinil molecule resides on the hydrophilic part of the Gelucire^®^ polymer. This is evident by the binding energy of -71.05 KJ mol^-1^ resulting from using MM bond dipole force field.

## 4. Discussion

Upon comparing Kawakita parameters between the two techniques, microwave samples manifested stronger granules (higher *P*_K_), whereas hot-melt samples manifested a powder with slightly higher compressibility (higher *a*).

When the fractional factorial design of the data was run using the Design Expert software, a quartic model was found to provide the best fit to the data with statistical significance at a *p*-value <0.05. Plots based on such a model (Figures 3-6) confirmed the previous finding in Tables 3 and 4 for microwave and hot-melted techniques, respectively. In other words, maximum *a* and *P*_K_ values were recorded at low Gelucire^®^ contents, more specifically, 3.4 % for microwave and around 5 wt. for hot-melt samples. Therefore, higher Gelucire^®^ content does not contribute to the optimum compression properties of the mixtures or the samples of low Gelucire^®^ content. Technically, the foregoing can be arguably justified by two main measured parameters at these aforementioned optimum Gelucire^®^ contents: tablet hardness and melting endotherms through DSC analysis.

Initially, tablets were fragile at all Gelucire^®^ contents except at 3.4 and 5 wt.% for microwave and hot-melt samples, respectively (Tables 3 and 4). This can be correlated to the formation of strong granules, as the Kawakita parameter (*P*_K_) indicates at these Gelucire^®^ contents. The *P_K_* values of the foregoing were numerically much higher than most other Gelucire^®^ contents.

For physical mixtures, compressibility becomes lower at higher Gelucire^®^ content. All compressed powders were physically weak, resulting in fragile tablets whose hardness cannot be measured. Physical properties were even worse since *P_K_* underwent a decrease at higher Gelucire^®^ content (Figure 7). The same can be said for compressibility, which dramatically decreased upon adding more Gelucire^®^ (Figure 8).

Crystallinity parameters for samples representing hot-melting and microwave processing techniques, along with samples representing pure modafinil raw material, were all initially compared without the addition of Gelucire^®^. In this manner, the influence of process types on changing the drug’s crystallinity can be well assessed. For this matter, samples that underwent hot-melting presented a slight increase in crystallinity, whereas, for microwave samples, crystallinity underwent a dramatic decrease. This change, either increase or decrease, is clearly evident by the three chosen crystallinity parameters in this work (crystallinity, *RC* and *RI*). In other words, any increase or decrease in crystallinity is followed by a concurrent increase or decrease in RC and *RI* values, respectively. It has to be noted that the foregoing statement is so far valid for samples comprising the drug alone without any presence of Gelucire^®^. Hot-melting and microwave processing, when Gelucire^®^ was present, showed a contrasting trend in crystallinity and increased Gelucire^®^ concentration. In this regard, from 5 to 15 %, hot-melt samples presented high crystallinity values, whereas microwave samples presented a drop at 3.4 and 10 % in Gelucire^®^ contents. Crystallinity in the former concentration was the lowest recorded drop for the modafinil-Gelucire^®^ complex subjected to microwave processing. At 20 % Gelucire^®^ content, it can be said that there is no difference between hot-melt and microwave samples with regard to crystallinity values. This means that the high content of Gelucire^®^ inside the complex profoundly reduces, almost removes, a process crystallinity influence. In other words, hot-melt and microwave processing likely have no remarkable effect on modafinil’s crystallinity at high Gelucire^®^ content.

It is important to mention that the microwave sample presented the lowest crystallinity value (42.47 %) at 3.4 % Gelucire^®^. As such, this, theoretically, indicates that the sample has the highest randomness in the distribution of atoms amongst all complexes investigated in this work. Such randomness implies that a more amorphous character in the complex manifests due to a possible interaction between modafinil and Gelucire^®^.

Concerning physical mixtures, variations of crystallinity with Gelucire^®^ content resemble that recorded for hot-melt samples. In other words, samples of low Gelucire^®^ content (5 % in this case) presented high crystallinity values, whereas samples comprising high content (*i.e.* 20 %) were similar to pure modafinil.

In a different context, it is important to bear in mind that variations in *RI* values upon changes in Gelucire^®^ concentrations were not coherently consistent, unlike the recorded changes in *RC* values (either in increasing or decreasing order) along with changing Gelucire^®^ concentrations. When applying changes in *RC* and *RI* values due to changes in Gelucire^®^ content, it was found that variations of *RC* parameter more closely resemble variations of crystallinity. In contrast, the *RI* parameter did not show a similar behavior. In fact, *RI* is far not consistent amongst the three parameters investigated in this work. For example, numerical outliers were recorded, specifically with hot-melt samples, when the Gelucire^®^ content increased from 5 to 15 wt.%. At these concentrations, the *RI* values were lower than pure modafinil's, unlike the behavior noticed with % crystallinity. In contrast, *RC* values for the same aforementioned concentrations were moderately higher than those of pure modafinil. Accordingly, crystallinity values calculated based on the area under crystalline peaks are more representative of the crystalline phase than peak intensities. Such a finding is suggested to be strong evidence -maybe solely confined to the current work - that x-ray peak intensities are not always aligned or in harmony with the material's crystallinity. This is in contrast to the general fact, which states that higher intensities suggest a higher degree of crystallinity due to the presence of a higher number of atoms capable of scattering X-rays [53]. It is suggested that such inconsistency in *RI* values is related to crystal size rather than the extent of arrangement/or randomness of atoms [54]. Accordingly, the low crystallinity at 3.4% Gelucire^®^ in the microwaved modafinil-Gelucire^®^ complex is more likely correlated to a larger crystal size than that at 10% and 20%. Nevertheless, the *RI* values of microwaved samples showed a similar trend to that of crystallinity when both were compared with their pure modafinil corresponding values. The foregoing remark is similarly valid for physical mixtures investigated herein at 3.4 and 20 wt.% Gelucire^®^.

For DSC analysis, the decrease in the melting point of modafinil is correlated to an increase in the free movements of atoms and/or to a change in the structural conformation of the drug [56]. It has to be further noted herein that the first peak of all complex samples represents the melting endotherm of Gelucire^®^. The intensity of this peak increased at higher Gelucire^®^ content in the complex. Regardless of any changes with this peak, it won’t be considered for investigation in the current work.

The fact that microwave-processed samples had lower melting points than samples subjected to hot-melt processing (Table 6) is correlated to the fact that the crystallinity values of microwave-processed samples are lower than those of hot-melt-processed samples (Table 5). Thus, there is more randomness in the distribution of atoms for microwave than hot-melt processed samples, rendering a higher drop in melting point for the former samples. The aforementioned behavior was mostly recorded for Gelucire^®^ content lower than 20 wt.%. It is suggested that at 20 wt.% Gelucire^®^, structural conformational changes were most likely the dominant explanation since crystallinity values for physical mixtures, hot melt, and microwave samples were almost similar.

Once again, the higher melting enthalpy (Figures 10 and 11, Table 6) generated for melting hot-melt samples than that generated for melting microwaved samples corresponds to the difference in crystallinity calculated for both samples. Generally, higher melting enthalpy corresponds to a high crystalline phase of a highly rigid structure with long-range order [57-60]. That can explain the high crystallinity values for hot-melt samples. In contrast, a decrease in melting enthalpy corresponds to a rise in the amorphous character of the modafinil-Gelucire^®^ complex. Such a decrease can be justified by the recorded drop in crystallinity values for microwaved samples. However, it has to be emphasized that not all DSC thermograms match with x-ray results. For example, the lowest melting enthalpy recorded for the microwave sample at 20 % Gelucire^®^ content had crystallinity values higher than that for samples at 3.4 and 10 wt.% Gelucire^®^. It is suggested that such non-harmony between DSC and X-ray is related to the fact that with regard to X-ray, samples were performed on a steady state, whereas with regard to DSC, samples were performed on a changeable state [61,62].

Another noticeable difference in the DSC of hot-melt and microwave samples was in the shape of a modafinil melting peak. The DSC of hot-melt samples presented a broad melting endotherm, increasing broadness and the Gelucire^®^ content (Figures 10 and 11). In contrast, all microwave samples showed sharp peaks in this regard. Physical mixtures of modafinil and Gelucire^®^ (10 wt.%) in Figure 11 presented no-interacting components since modafinil’s endothermic peak was sharp and its melting point did not undergo a change. Theoretically, there are various justifications behind endotherm peak broadening. Some are related to the size distribution of crystallites, and others to the fact that temperature can be too high for recrystallisation of melting crystals. The closest reasonable justification is related to the large heat needed for melting [63,64]. Accordingly, the higher melting enthalpy, as calculated in Table 6 and the higher melting points for hot-melt samples compared to microwave samples can all justify the extensive broadening of hot-melt melting endotherm.

Interestingly, at 3.4 % Gelucire^®^ content for microwave processing, which showed optimum compression performance, samples of the same Gelucire^®^ content (3.4 %) showed the lowest crystallinity for microwave and hot-melt samples (lowest crystallinity and *RC*). This is advantageous since lower crystallinity enhances powder flowability, compressibility, and tablet compatibility [65]. When the x-ray diffraction patterns of the pure drug and all microwave samples (Figure 9) are put together for comparison, it can be visually noticed that microwave patterns are different amongst each other and the pure drug. The number and intensity of diffraction peaks decreased upon decreasing the Gelucire^®^ content. That explains the low *RC* and *RI* values for complex samples that result from reduced Gelucire^®^ content. On the other hand, for hot-melt samples, sharper peaks with high intensities were noticed at the 5 wt.% sample, indicating a high *RI* value when compared to the 15 wt.% Gelucire^®^ sample.

FTIR has provided more evidence on the reduction of crystallinity, especially in microwave samples. The first proof in this regard was noticed on the irregular baseline that appeared with noise and pulsed shape. The second evidence suggests the appearance of three new peaks between 1500 and 1550 cm^-1^. Theoretically, the foregoing range represents an O-H stretching vibration mode, which could result from a possibly generated re-arrangement of the neighboring crystal lattice. In other words, a hydrophobic complexation between the oxygen and hydrogen from either of the two molecules is more likely. Accordingly, molecular modelling becomes important to perform at this stage.

The calculated binding energies using all MM force fields suggest that the modafinil/Gelucire^®^ complex formation is thermodynamically favored (Table 8). Based on these binding energies, it is obvious that the most stable complex was for the model 2 complex, in which the modafinil molecule resides on the hydrophilic part of the Gelucire^®^ polymer. This is evident by the binding energy of -71.05 kJ mol^-1^ resulting from using the MM bond dipole force field.

From an MM perspective according to model 2 whereby both modafinil/(hydrophilic part) Gelucire^®^ interact via non-classical hydrogen bonding, the chemical affinity of the hydrophilic part of Gelucire^®^ has been reported to take place towards carbonyl oxygen such as hydrogen bond complexation with the ketone group of curcumin in the curcumin/gelucire complex [66]. A dipole-dipole interaction was further reported between the carbonyl group of tolbutamide and Gelucire^®^ [67]. Similarly, numerous examples were reported [68-70] in the same regard; however, objectives were all aligned towards increasing drug solubility by lowering crystallinity and drug melting point [71].

## Conclusions

Shortcomings in modafinil powder and tablet properties can be overcome through microwave processing of modafinil-Gelucire^®^ combinations. Hot-melt processing of modafinil with Gelucire^®^ was concurrently compared. However, it did not prove to be as effective as microwave processing. Optimum Gelucire^®^ concentration (3.4 %) was found to cause optimum powder compression behavior due to the lowest recorded drug crystallinity manifested through x-ray diffraction and DCS analysis. Molecular modelling suggested a dipole-dipole interaction by binding the hydrophilic part of Gelucire^®^ with the carbonyl oxygen of modafinil. Such interaction justifies the low complex crystallinity and, thus, the improved powder and tablet properties.
